# Outcomes of patients with different lepidic percentage and tumor size of stage I lung adenocarcinoma

**DOI:** 10.1111/1759-7714.14477

**Published:** 2022-06-09

**Authors:** Chia Liu, Lei‐Chi Wang, Hui‐Shan Chen, Yi‐Chen Yeh, Po‐Kuei Hsu, Chien‐Sheng Huang, Chih‐Cheng Hsieh, Han‐Shui Hsu

**Affiliations:** ^1^ Division of Thoracic Surgery, Department of Surgery Taipei Veterans General Hospital Taipei Taiwan; ^2^ Department of Pathology and Laboratory Medicine Taipei Veterans General Hospital Taipei Taiwan; ^3^ Department of Health Care Administration Chang Jung Christian University Tainan City Taiwan; ^4^ School of Medicine National Yang‐Ming Chiao Tung University Taiwan

**Keywords:** adenocarcinoma of lung, invasive size, lepidic component, part‐solid tumor, TNM staging system

## Abstract

**Background:**

To evaluate the long‐term outcomes after surgical resection for stage I lung adenocarcinoma based on the percentage of lepidic component (LC) and invasive tumor size (IS).

**Methods:**

The clinicopathological characteristics of 1049 patients with stage I lung adenocarcinoma who underwent surgery between 2006 and 2016 were retrospectively reviewed. Tumors were categorized into groups: A (LC ≥ 50%) and B (LC < 50%). Groups A0 and A1 consisted of minimally invasive adenocarcinomas (MIA) and other lepidic‐predominant invasive adenocarcinomas, respectively. Group B was categorized into B1 (IS ≤ 1 cm), B2 (1 < IS≤2 cm), and B3 (2 < IS≤3 cm) by invasive tumor size and divided into subgroups (B1[lep+]/[lep−], B2[lep+]/[lep−], and B3[lep+]/[lep−]) according to the presence[lep+] or absence[lep−] of LCs. Cumulative incidence of recurrence (CIR) and cancer‐specific survival (CSS) were examined.

**Results:**

LC decreased with increasing IS. Only 24 (8.5%) tumors in group A had an IS >1 cm. 10‐year CIR and CSS were 15.2% and 86.0%. LC and IS were found to be independent predictors of CSS. Patients in group A had 1.4% 10‐year CIR and 100% 10‐year CSS. In group B, a significantly higher CIR and worse CSS were observed as IS increased (*p* < 0.001), but LC was not a predictor for CSS (*p* = 0.593). No significant differences in CIR or CSS were found in presence of LC or not when LC < 50% (B1[lep+]/[lep−], B2[lep+]/[lep−], and B3[lep+]/[lep−]: *p* = 0.36/0.48, *p* = 0.82/0.94, and *p* = 0.90/0.37, respectively).

**Conclusions:**

LC≥50% tumors demonstrated excellent prognosis regardless of IS. The outcomes of LC < 50% tumors were well predicted by IS, corresponding to the T‐staging system. The predictive value of LC for prognosis became insignificant.

## Graphical abstract

Surgical outcomes of stage I lung adenocarcinoma with different lepidic percentage and invasive tumor size

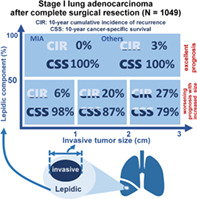

## INTRODUCTION

Adenocarcinoma is the most prevalent histological non‐small cell lung cancer (NSCLC), especially in Asia.^1^ It is a heterogeneous disease with a wide spectrum of prognoses. With the increasing use of low‐dose computed tomography (CT) for screening after the National Lung Screening Trial^2^ and improvements in image resolution, more tumors ≤3 cm or containing ground‐glass opacity (GGO) components, especially part‐solid nodules,^3^ were detected and categorized as stage I lung adenocarcinoma on pathological analysis after resection. Part‐solid adenocarcinomas containing different ratios of noninvasive lepidic components found with pathological analysis were traditionally classified into different T stages, mainly based on total tumor size. In the eighth edition of the American Joint Commission on Cancer Tumor–node–metastasis (TNM) staging system for NSCLC, the invasive size of the tumor (IS), the size excluding lepidic component (LC), became a new indicator for T stage because of its better predictive value for prognosis.^4^, ^5^, ^6^, ^7^, ^8^

For early‐stage lung adenocarcinomas, the behavior of tumors can no longer be precisely evaluated by invasive size alone. Different percentages of LC in part‐solid nodules may imply distinctive invasiveness and play an important role in patient outcomes. For example, the patients with adenocarcinoma in situ (AIS) or minimally invasive adenocarcinoma (MIA) had nearly 100% freedom from cancer‐specific death or recurrence.^5^, ^9^, ^10^ In previous studies,^11^, ^12^, ^13^, ^14^ lepidic‐predominant invasive adenocarcinoma also carried more desirable prognosis than adenocarcinomas with other predominant subtypes, and led to more than 90% recurrence‐free survival (RFS).

However, influence of LC on the long‐term prognosis of stage I lung adenocarcinoma was still controversial.^5^, ^15^, ^16^ Accordingly, this study was conducted to evaluate the long‐term outcomes of stage I lung adenocarcinoma with different LC and IS after complete surgical resection, using cumulative incidence of recurrence (CIR) and cancer‐specific survival (CSS) as indicators.

## METHODS

### Patients

**FIGURE 1:**
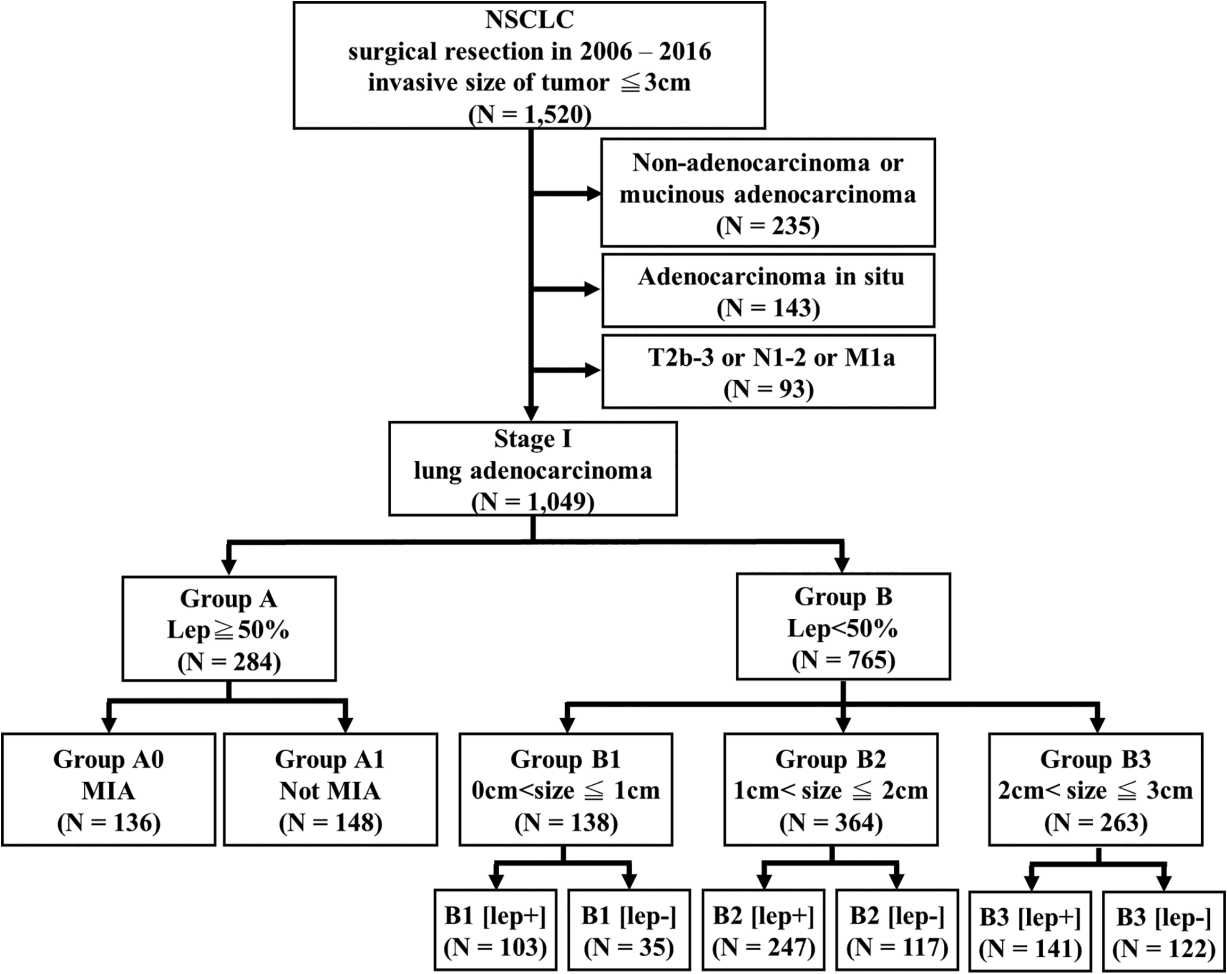
Flow diagram of patients selected and included in the present study

A total of 1520 patients with solitary NSCLC sized ≤3 cm on invasive focus underwent complete resection at Taipei Veteran General Hospital between 2006 and 2016. Excluding 235 patients with nonadenocarcinoma, mucinous adenocarcinoma, or fetal adenocarcinoma, 143 patients with AIS, and 93 patients with nodal positive or metastatic disease, the present study enrolled 1049 patients with pathologically proven stage I lung adenocarcinoma (Figure 1). Clinical demographic characteristics were recorded for further analysis, including age, sex, Eastern Cooperative Oncology Group (ECOG) performance status, Charlson comorbidity index, preoperative lung function, smoking history, symptomatic presentation, preoperative serum carcinoembryonic antigen (CEA) level, surgical procedure, and adjuvant chemotherapy. The study protocol was approved by the Institutional Review Board of Taipei Veterans General Hospital, and the requirement for signed informed consent from the patients was waived (Approval no. 2021‐08‐005B).

### Preoperative staging workup and lymph node dissection

The preoperative staging workup includes a thin‐section contrast chest CT scan with multidimensional slicing and reconstruction into axial, coronal, and sagittal views. Whole body bone scan or positron emission tomography (PET) scan, and brain survey (CT or magnetic resonance imaging [MRI]) for distant metastasis were also performed to determine clinical stages. Mediastinal evaluation included mediastinoscopy, endobronchial ultrasound fine‐needle aspiration, intraoperative lymphadenectomy, or preoperative PET scan. Patients underwent either radical mediastinal lymphadenectomy (the majority) or mediastinal node sampling, according to the surgeon's preference.

### Pathological examination

The pathological stage was diagnosed using the eighth TNM system for lung cancer.^17^ Elastic staining was performed in tumor sections when the status of visceral pleural invasion was indeterminable with hematoxylin and eosin staining.^18^ Angiolymphatic invasion was defined as vascular invasion or lymphatic permeation. For the description of histopathological components, the occupancy of LC in the total tumor area was measured and recorded in 5% increments.^19^ The LC, and total and invasive tumor size on pathology were reviewed by two specialized thoracic pathologists (L‐C Wang and Y‐C Yeh). IS was measured under a microscope using a ruler or calculated by multiplying the total tumor size by the percentage of invasive components.^5^

Patients were then categorized into groups A (LC ≥ 50%) and B (LC < 50%) according to LC. Groups A0 and A1 consisted of minimally invasive adenocarcinomas (MIAs) and other lepidic‐predominant invasive adenocarcinomas, respectively. Patients in group B were categorized into groups B1 (0 < size≤1 cm), B2 (1 < size ≤2 cm), and B3 (2 < size≤3 cm) according to the invasive tumor size and divided into subgroups (B1[lep+]/[lep−], B2[lep+]/[lep−], and B3[lep+]/[lep−]) according to the presence[lep+] or absence[lep−] of LCs.

### Follow‐up after surgery

Chest radiography was performed every 3 months for the first 2 years after surgery, every 6 months from the third to fifth year, and annually thereafter. Chest CT scans were performed every 6 months for 2 years and then annually. Patients who did not visit this hospital in the past year were called to confirm their survival status. Those who had been out of contact for 1 year or longer were defined as lost to follow‐up. Except 76 patients lost to follow‐up, all the other patients had been followed up with until April 30, 2021 (follow‐up rate 93.6%).

### Analysis of recurrence and survival

Cumulative incidence of recurrence (CIR) and cancer‐specific survival (CSS) were examined. Recurrence was confirmed using tissue biopsy or clinically determined by a multidisciplinary lung cancer committee. Patients with synchronous unresected GGOs and metachronous tumors were excluded to distinguish between ipsilateral and contralateral recurrences at the beginning of the study.^20^ Patterns of failure were defined as local recurrence for any recurrent disease within the ipsilateral hemithorax, mediastinum, or supraclavicular lymph nodes, and as distant metastasis for all other sites of recurrence.

CSS was defined as the interval between the date of surgical resection and date of death due to lung cancer. Observations were censored at the last follow‐up session on patients who were still alive or on those who died from diseases other than lung cancer.

### Statistical analysis

Patient characteristics among the different groups were compared using the Pearson's Chi‐square test for categorical variants. The distribution of continuous variables was first analyzed using the Shapiro–Wilk test of normality, and then an independent ANOVA or Kruskal‐Wallis test was utilized according to the results. Tumors of different LC and IS are presented on a scatter plot with a locally estimated scatterplot smoothing (LOESS) smoothed trend line. The risk of recurrence (CIR) was evaluated using the cumulative incidence function. Hazard ratios (HRs) and 95% confidence intervals (CIs) were calculated using the Fine & Gray model. Comparison of CIR between the groups was performed using Gray's test.^21^ Univariate analysis for the prognostic factors of CSS was performed using the Cox regression method. Multivariate analysis of prognostic factors with *p* ≤ 0.1 was performed. The results are expressed as odds ratios with 95% CIs. *p* < 0.05 was considered statistically significant. CSS curves were estimated using Kaplan–Meier method. Differences in survival between strata were examined using the log‐rank test. Statistical analyses were performed using SPSS Statistics Version 25.0 for Windows (IBM Corporation) and R (http://www.R-project.org/) version 4.1.1.

## RESULTS

### Patient characteristics

A total of 1049 patients with stage I nonmucinous adenocarcinoma were included. The clinicopathological factors are summarized in Table 1. The median age of patients was 62 years of which 441 (42.0%) were male. Around one‐fourth of the patients were smokers (*n* = 273, 26.0%), 263 presented with symptoms before diagnosis (25.1%), and 105 (10%) had elevated serum CEA levels before surgery. Most patients (*n* = 783; 74.6%) underwent at least lobectomy, and the remaining 266 (25.4%) underwent sublobar resection. The median whole tumor size and IS were 1.8 and 1.4 cm, respectively. The median of LC was 20%. There were 284 patients with LC ≥50% in group A, and 765 patients in group B (LC <50%). Compared with group B patients, group A patients were relatively younger, predominately female, less symptomatic, and had fewer smokers. In group B, patients with larger tumors tended to be symptomatic, had a smoking history, and underwent lobectomy at least.

**TABLE 1:** Characteristics in patients with different percentage of lepidic component in lung adenocarcinoma

Patient characteristics	All	Lep ≥ 50%	Lep < 50%	*p*‐value
	(*N* = 1049)	(*N* = 284)	(*N* = 765)	
	Number (%)	Number (%)	Number (%)	
	Median (IQR 25%–75%)	Median (IQR 25%–75%)	Median (IQR 25%–75%)	
Age (year)	62 (55–70)	60 (52–66)	63 (56–71)	<0.001^a^
Gender (male)	441 (42.0)	104 (36.6)	337 (44.1)	0.03^b^
ECOG				<0.001^b^
0	655 (62.4)	209 (73.6)	446 (58.3)	
1	386 (36.8)	75 (26.4)	311 (40.7)	
2	8 (0.8)	0 (0)	8 (1)	
Charlson comorbidity index	2 (1–3)	2 (1–3)	2 (1–3)	0.32^a^
Smoking history (smoker)	273 (26.0)	57 (20.1)	216 (28.2)	0.007^b^
Symptomatic	263 (25.1)	50 (17.6)	213 (27.8)	0.001^b^
Elevated CEA level	105 (10.0)	20 (7.0)	85 (11.1)	0.05^b^
Operative method				<0.001^b^
Wedge	215 (20.5)	94 (33.1)	121 (15.8)	
Segmentectomy	51 (4.9)	27 (9.5)	24 (3.1)	
Lobectomy	779 (74.3)	162 (57.0)	617 (80.7)	
Bilobectomy	3 (0.3)	1 (0.4)	2 (0.3)	
Pneumonectomy	1 (0.1)	0 (0)	1 (0.1)	
T stage				<0.001^b^
TMI	136 (13.0)	136 (47.9)	0 (0)	
T1a	138 (13.2)	49 (17.3)	89 (11.6)	
T1b	139 (13.3)	7 (2.5)	132 (17.3)	
T1c	67 (6.4)	0 (0)	67 (8.8)	
T2a	569 (54.2)	92 (32.4)	477 (62.4)	
Whole tumor size (cm)	1.8 (1.3–2.5)	1.4 (1.0–2.0)	2.0 (1.5–2.5)	<0.001^a^
Invasion size (cm)	1.4 (0.7–2.1)	0.4 (0.2–0.6)	1.7 (1.2–2.3)	<0.001^a^
Lepidic component (%)	20 (0–50)	70 (60–90)	10 (0–20)	<0.001^a^
Pleural invasion (present)	568 (54.1)	92 (32.4)	476 (62.2)	<0.001^b^
Angiolymphatic invasion (present)	113 (10.8)	2 (0.7)	111 (14.5)	<0.001^b^
Number of lymph nodes harvested	15 (11–22)	13 (8–19)	16 (12–23)	<0.001^a^
Adjuvant chemotherapy	244 (23.3)	31 (10.9)	213 (27.8)	<0.001^b^

^a^ Mann–Whitney U test.

^b^ Chi square test.

Abbreviation: ECOG, Eastern Cooperative Oncology Group.

### Distribution of tumors with different lepidic component ratio, invasive size and subgrouping

**FIGURE 2:**
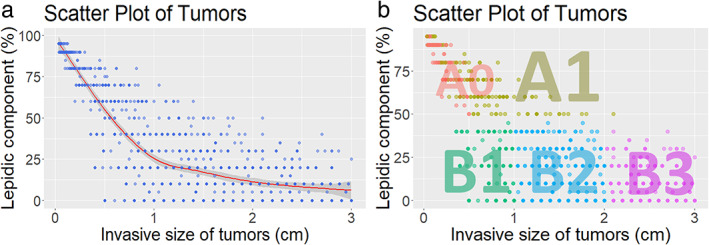
(a) Scatter plot of tumors according to the percentage of lepidic component and invasive tumor size. (b) Grouping in the current study

Figure 2a shows the distribution of LCs in the different ISs in this cohort. In stage I lung adenocarcinoma, locally weighted regression showed a decreasing LC with an increase in IS. When the IS exceeded 1 cm, only 24 patients (8.5%) had a tumor with ≥50%; when the IS exceeded 1.5 cm, no tumor was found to have LC ≥ 50%. For further grouping analysis, group A was divided into A0 (MIA, *n* = 136) and A1 (other than MIA, *n* = 148). Group B was further divided into groups B1 (0 < IS≤1 cm, *n* = 138), B2 (1 < IS≤2 cm, *n* = 364), and B3 (2 < IS≤3 cm, *n* = 263; Figure 2b). The median of whole tumor size and IS was 1 and 0.8 cm in B1, 1.8 and 1.5 cm in B2 and 2.6 and 2.5 cm in B3, respectively.

### Recurrence

The median follow‐up period was 86.1 months (interquartile range, IQR: 62.8–117.0), during which the tumor recurred in 148 patients (14.1%), including 60 (5.7%) with local recurrence only, 35 (3.3%) with distant recurrence only, and 53 (5.1%) with local and distant recurrence (Table 2). There was no recurrence in group A0, and only four (9.0%) in group A1 (two local, one distant, and one local with distant recurrence). Two of the patients with distant metastasis were staged pT2aN0M0 based on pleural invasion status. Patient 1 was a woman diagnosed with lung adenocarcinoma at the age of 73. The total tumor size and invasive tumor size were 3.0 and 1.5 cm, and composed of lepidic (50%), acinar (30%), micropapillary (10%) and solid (10%) components. Brain metastasis occurred 30 months after left S6 segmentectomy. Patient 2 was a man diagnosed with lung adenocarcinoma at the age of 79. The total tumor size and invasive tumor size were 2.7 and 1.4 cm, composed of lepidic (50%), acinar (30%), and micropapillary (20%) components. Bilateral lung metastasis occurred 34 months after RUL wedge resection. In group B, the rate of recurrence increased significantly with increasing size (B1: 5.1%, with 4.3% local, and 0.7% local and distant; B2: 18.4%, with 6.0% local, 4.7% distant, and 7.7% local and distant; B3: 26.6%, with 11.4% local, 6.5% distant, and 8.7% local and distant, *p* < 0.001).

**TABLE 2:** Pattern of first recurrence after surgery

Patient characteristics	All	A0/A1	B1	B2	B3	*p‐*value
	(*N* = 1049)	(*N* = 284)	(*N* = 125)	(*N* = 343)	(*N* = 301)	
Total recurrence No. (%)	148 (14.1)	4 (1.4)	7 (5.1)	67 (18.4)	70 (26.6)	*p* < 0.001
Pattern of recurrence						*p* < 0.001
Local alone (%)	60 (5.7)	2 (0.7)	6 (4.3)	22 (6.0)	30 (11.4)	
Distant alone (%)	35 (3.3)	1 (0.4)	0	17 (4.7)	17 (6.5)	
Local and distant (%)	53 (5.1)	1 (0.4)	1 (0.7)	28 (7.7)	23 (8.7)	

*Note*: A0/A1 = Lep ≥ 50%; B1 = Lep < 50%, 0 < size ≤ 1 cm; B2 = Lep < 50%, 1 < size ≤ 2 cm; B3 = Lep < 50%, 2 < size ≤ 3 cm.

Abbreviations: Lep, lepidic component.

**FIGURE 3:**
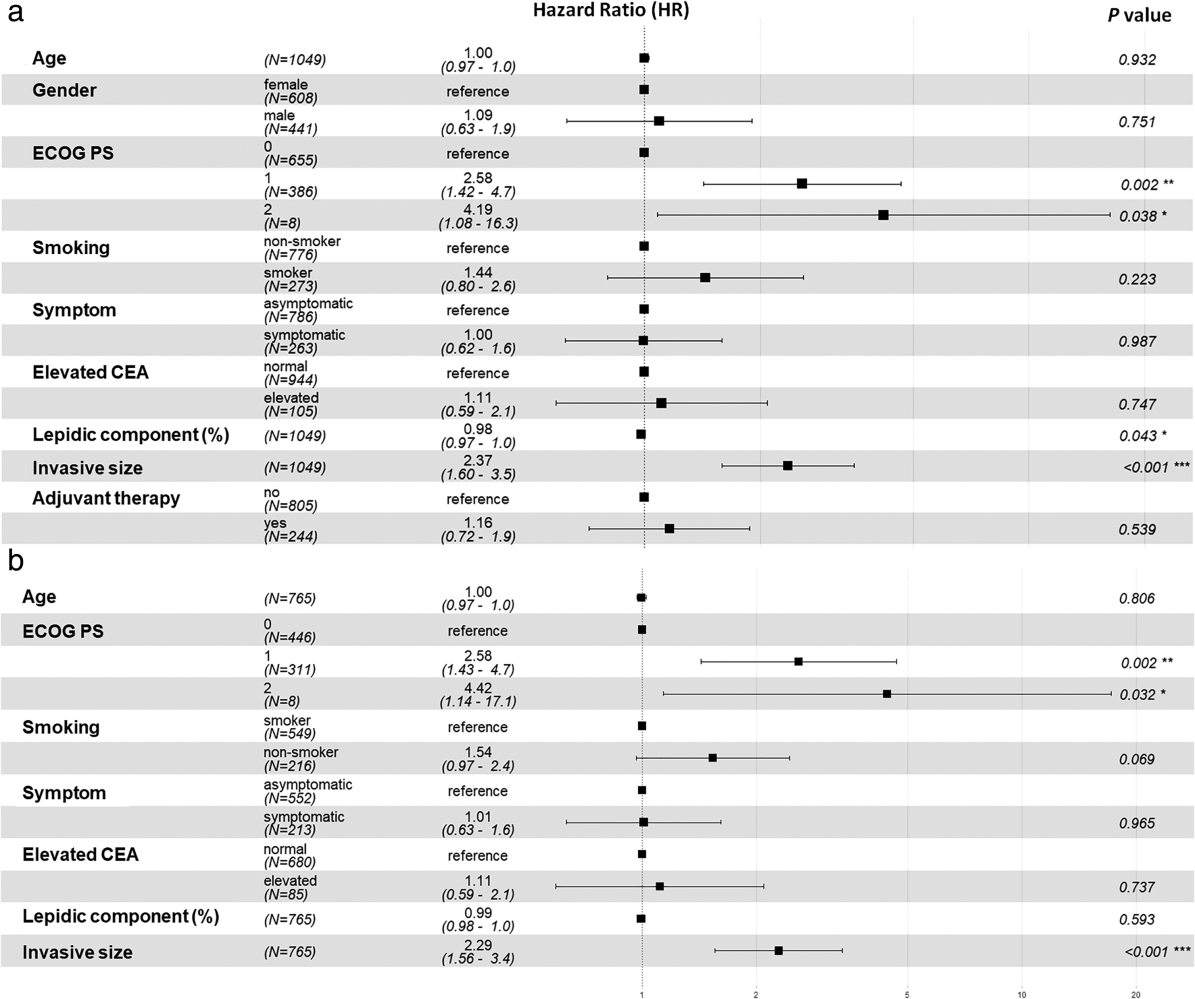
Multivariable Cox regression analysis demonstrated by forest plots in the whole cohort and group B

In the overall cohort, the 10‐year CIR was 15.2% (95% CI: 12.8–17.7) and significantly lower in group A (1.4%: 0–2.9) than in group B (19.8%: 16.8–22.9, *p* < 0.001). In group A, there was no significant difference between groups A1 (2.7%: 0.03–5.5) and A0 (0) (*p* = 0.056). In group B, the 10‐year CIR increased significantly with invasive tumor size (B3: 27.0%, 21.4–32.6; B2: 19.8%: 15.3–24.2; B1, 6.4%: 1.2–11.6; B1 vs. B2: p < 0.001, B2 vs. B3: *p* = 0.02; Figure 3a).

### Survival analysis

**FIGURE 4:**
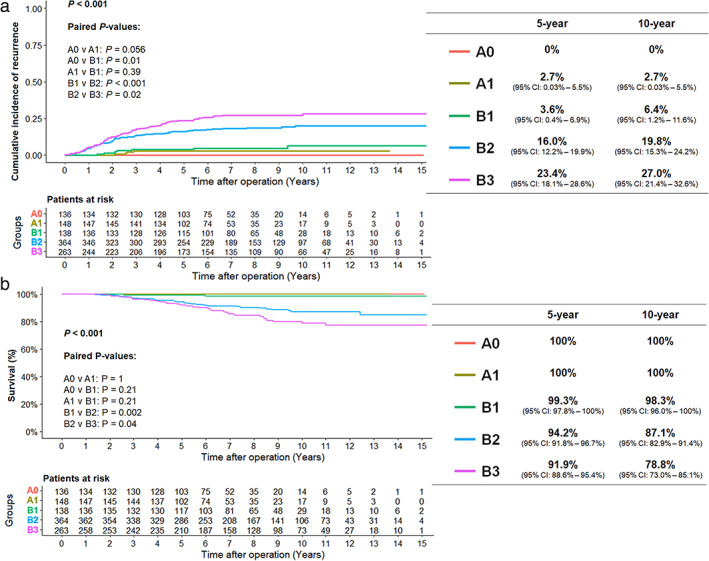
(a) The cumulative incidence of recurrence and (b) cancer‐specific survival in groups

Eighty‐two cancer‐specific deaths occurred during the follow‐up period. ECOG performance status, lower LC (*p* = 0.043), and larger IS were identified using multivariable Cox regression analysis as independent risk factors for CSS in the whole cohort. In group B, only ECOG performance status and larger IS on pathological analysis were independent risk factors for CSS. LC was not an independent prognostic factor in patients with LC less than 50% (*p* = 0.593, Figure 4).

The 10‐year CSS was 86.0% in the overall cohort (95% CI: 83.0–89.1), 100% in group A, and 86.0% (83.0–89.1) in group B. It reduced significantly with the growth of IS (B1: 98.3% [96.0–100], B2: 87.1% [82.9–91.4], and B3: 78.8% [73.0–85.1]; B1 vs. B2: *p* < 0.002, B2 vs. B3: *p* = 0.04; Figure 3b). There was no significant difference in CSS between groups A0, A1 and B1 (A0 vs. A1: *p* > 0.99, A1 vs. B1: *p* = 0.21).

### Role of lepidic ratio in tumors with less than 50% lepidic component

**FIGURE 5:**
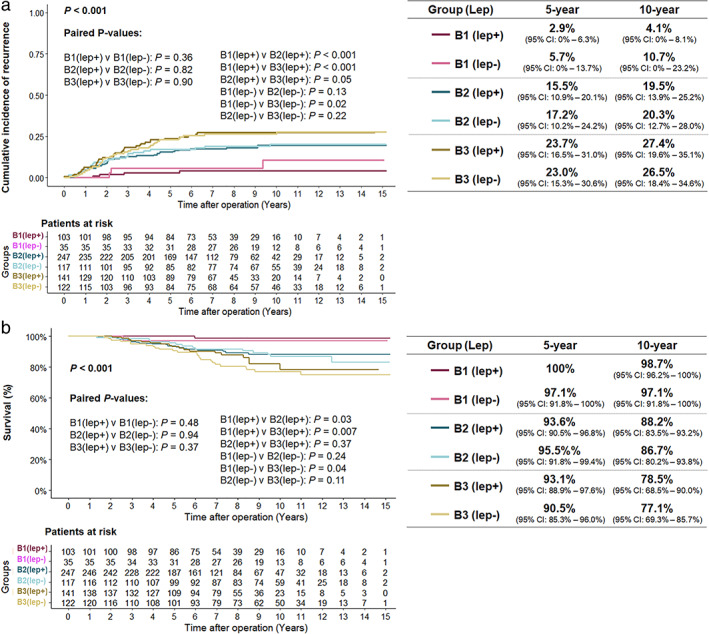
The subgrouping of lepidic (+) and lepidic (−) in group B. (a) The cumulative incidence of recurrence and (b) the cancer‐specific survival

While the ratio of LC was not an independent predictor of CSS in group B, we further divided these three subgroups by the presence or absence of LC. There was no significant difference in the CIR and CSS between groups B1[lep+]/[lep−], B2[lep+]/[lep−], and B3[lep+]/[lep−]: *p* = 0.36/0.48, *p* = 0.82/0.94, and *p* = 0.90/0.37, respectively, Figure 5).

## DISCUSSION

Our study on the distribution of LC ratio in different invasive tumor sizes in patients with stage I adenocarcinoma of the lung who underwent surgical intervention demonstrated a decrease in LC with an increase in IS in the overall cohort studied. Extremely few tumors presented LC > 50% with IS >1 cm, and no tumor was found to have LC >50% with IS >1.5 cm. Among the CIR and CSS analyzed, tumors with LC ≥50% had excellent prognosis. In contrast, in group B, the LC ratio was not a predictor of CSS in the multivariate analysis (*p* = 0.593), and the outcomes were well predicted by IS corresponding to the current T staging system.

A previous study found a correlation between lepidic‐predominant invasive tumors with smaller total tumor size (*p* = 0.027) and lower T stage (*p* < 0.001), compared with nonlepidic‐predominant tumors.^5^ Some observational studies also compared the longest doubling time of tumors between lepidic‐predominant tumors and other predominant histology subtypes.^22^, ^23^ In the present study, the relatively slow‐growing rate of LC might explain the reason its proportion decreased rapidly with the emergence of invasive components. Another possible reason might also be that, as suggested by current guidelines, a part‐solid tumor would be resected if the pure GGO tumor underwent a solid change.^24^

The patients in group A had excellent prognosis, with 100% CSS and 1.4% CIR. None of the patients in the A0 group (MIA) experienced tumor recurrence. Only four out of 148 patients experienced recurrence in group A1, and all had T2a disease based on pleural invasion. Two patients underwent segmentectomy and the other two underwent wedge resection and lobectomy. There was no recurrence in the cohort of patients with pT1N0M0 and LC > 50%. Kadota et al. demonstrated similar results in patients with stage I lung adenocarcinomas. Their study revealed 0% 5‐year CIR for 84 tumors with LC ≥50% after lobectomy or sublobar resection.^5^ Moon et al. also reported a 90% 3‐year RFS for patient with LC ≥50% tumors.^15^ Together, the outcomes of this group of patients were satisfactory, regardless of invasive tumor size.

The current definition of MIA includes invasive size ≤0.5 cm and total tumor size ≤3 cm. A Japanese case series reported 18 patients with lepidic‐predominant lung adenocarcinoma with invasive size ≤0.5 cm but total size >3 cm, and yielded 100% 5‐year CSS. The authors proposed withdrawing the restriction of total tumor size based on the criteria of MIA.^25^ We reviewed 11 patients in group A with a total tumor size above 3 cm (range: 3.1– 4.2 cm). Only one patient experienced pleural seeding 25 months after segmentectomy. None of the other 10 patients experienced recurrence after lobectomy. Among 148 patients in group A who had invasive sizes between 0.5 and 3 cm, no cancer‐specific death was recorded with only four recurrences. The results support the possibility of extending the definition of MIA to this special group. However, Naito et al. reported significantly lower 5‐year RFS of lepidic‐predominant adenocarcinoma of lung in patients with total tumor size >3 cm than in those with total tumor size <3 cm (73% vs. 93%, *p* < 0.01).^26^ Further investigation is needed to determine the prognosis in this small group of patients.

In the present study, LC was not an independent predictor of CSS in group B. In this group of patients, the IS determined the prognosis, a result corresponding to the current TNM staging system. Zhu et al. claimed that the presence of LC itself was sufficient to define a subgroup of patients with good prognosis, regardless of the invasive size or lepidic ratio in stage I lung adenocarcinoma.^16^ In contrast, our present study demonstrated no significant difference in CIR and CSS between the B1[lep+]/[lep−], B2[lep+]/[lep−], and B3[lep+]/[lep−] groups. In other words, LC ratio or the presence of lepidic components or not might become insignificant when the lepidic ratio is <50%.

There are several limitations to our study. First, this study was conducted retrospectively at a single institute. Therefore, it was difficult to avoid patient bias to achieve a normal distribution of patient characteristics, as well as strong statistical power. For example, only 24 patients had tumors with LC ≥ 50% and invasive size >1 cm. Low statistic power was expected if we compared this group of patients directly with those having LC < 50% but the same invasive size of tumor. Second, inconsistencies in the measurement of invasive size and LC might have existed among pathologists. Third, selection bias might have occurred due to relatively low event rates in some subgroups. Fourth, this was a female and nonsmoker cohort consisting of all Asian patients. These results might not be applicable to other populations. Last, there was no information of tumor spread through air spaces before 2014. Therefore, we could not include this factor in the present study. However, the present study still had the advantage of having a sufficient number of patients with part‐solid adenocarcinoma so that long‐term follow‐up could easily be completed to achieve long‐term outcomes.

In conclusion, in patients with stage I lung adenocarcinoma, LC decreased with an increase in IS. The importance of LC and IS in prognosis changes with a decrease in the lepidic ratio. Patients with LC ≥50% tumors had excellent prognosis after surgical resection regardless of size, even with IS >5 mm or total tumor size >3 cm. In this group of patients, the current TNM stage might not have an effect on long‐term outcomes. Among the patients with LC < 50% tumors, IS was an independent prognostic predictor for CSS, corresponding to the current TNM staging system, but the predictive value of LC became insignificant.

## CONFLICT OF INTEREST

The authors confirm that there are no conflicts of interest.
